# Clinical efficacy and safety of hypernormal shortened door to needle time (DNT) plus individualized low-dose alteplase therapy in treating acute ischemic stroke

**DOI:** 10.12669/pjms.324.9518

**Published:** 2016

**Authors:** Mei Zheng, Hongyan Lei, Yansen Cui, Daiqun Yang, Liquang Wang, Ziran Wang

**Affiliations:** 1 Mei Zheng, Department of Emergency, Linyi People’s Hospital, Linyi 276000, China; 2 Hongyan Lei, Department of Emergency, Linyi People’s Hospital, Linyi 276000, China; 3 Yansen Cui, Department of Emergency, Linyi People’s Hospital, Linyi 276000, China; 4 Daiqun Yang, Department of Emergency, Linyi People’s Hospital, Linyi 276000, China; 5 Liquang Wang, Department of Emergency, Linyi People’s Hospital, Linyi 276000, China; 6 Ziran Wang, Department of Emergency, Linyi People’s Hospital, Linyi 276000, China

**Keywords:** Hypernormal shortened DNT, Alteplase, Acute ischemic stroke, Efficacy and safety

## Abstract

**Objective:**

This study aims to observe the clinical efficacies of hyper-early low-dose alteplase thrombolysis in treating acute ischemic stroke (AIS).

**Methods:**

Two hundred twenty AIS patients were randomly divided into group A (90 cases), group B (90 cases), and group C (40 cases). The National Institutes of Health Stroke Scale (NIHSS) scores, mRS score-evaluated prognosis, intracranial hemorrhage, and mortality of the three groups were observed before and after the treatment.

**Results:**

The NIHSS scores of the three groups were significantly reduced after the treatment (P<0.05), among which the NIHSS score of group A was the lowest (P<0.05); and the difference between group B and C was not significant (P>0.05). The incidence of such complications as cerebral hemorrhage in the three groups was low, and there was no significant difference among the groups (P>0.05). The modified Rankin Scale (mRS)scores of the three groups showed that group A had much better prognosis than group B and C, while the difference between group B and group C was not significant.

**Conclusions:**

The hyper-early low-dose alteplase thrombolysis was safe and effective in Acute ischemic stroke (AIS).

## INTRODUCTION

Stroke is a common clinical cerebrovascular disease characterized by high incidence, high morbidity, high recurrence, and high mortality, and it seriously affect patients’ quality of life.^1^-^3^ A large number of clinical studies had found that stroke incidence was rising, and gradually became an important cause of clinical death.^4^,^5^ Numerous studies had confirmed that early intravenous thrombolysis was an effective method to treat acute ischemic stroke (AIS), and the earlier the thrombolytic therapy, the greater the likely benefits for the patients, and the risk of bleeding transformation would also be reduced.^6^

However, the current thrombolysis guidelines towards ischemic stroke in all countries request various types of inspections before thrombolytic therapy, which sometimes might increase the intra-hospital waiting time due to less smooth flowsheet, and this would undoubtedly reduce the thrombolytic effects and increase the risk of bleeding transformation. How to use a variety of measures to carry out the individualized thrombolytic therapy as early and quickly as possible and to shorten the treatment time has become a research hotspot. Alteplase (rt-PA) is a recombinant tissue-type plasminogen activator, by binding to fibrin, it could activate and transfer the plasminogen into plasmin, thus dissolving thrombus.^7^-^9^ rt-PA is the drug currently recommended for stroke in the AIS treatment guidelines of different countries.^10^-^12^ Because of the differences in the races, physical conditions, or other aspects among different countries, the application dose of rt-PA still needs to be further explored in clinical.

In this study, 220 AIS patients admitted in our hospital had individualized thrombolytic therapies, through observing such problems as how to shorten the medication time and reduce the dosage, etc., aiming to explore the clinical efficacy and safety of hypernormal shortened door to needle time (DNT) plus low-dose alteplase therapy in treating AIS, and to provide evidence for clinical studies.

## METHODS

This retrospective study included 220 AIS patients admitted into the department of emergency of our hospital from June 2012 to February 2014, including 139 males and 81 females, aged 37-85 years, with the mean age as 62.2±11.8 years. All patients had the following clinical features: hospitalized within 4.5 hours after onset; without intracranial hemorrhage or low density shadow by computed tomography (CT) scanning, or image of early cerebral infarction (such as the sulcus disappeared), or with asymptomatic or non-sign lacunar cerebral infarction; with clear nervous system involvement; with the National Institutes of Health Stroke Scale (NIHSS) score as 4-22 points. The patients were randomly divided into group A (90 cases), group B (90 cases), and group C (40 cases). This study was conducted in accordance with the declaration of Helsinki. This study was conducted with approval from the Ethics Committee of Linyi People’s Hospital. Written informed consent was obtained from all participants.

### Exclusion criteria

1) with the signs of intracranial hemorrhage and subarachnoid hemorrhage; 2) with severe hypertension, systolic blood pressure ≥185 mmHg or diastolic blood pressure ≥110mmHg, or could not achieve the target values after the treatment; 3) stroke accompanied with epilepsy; 4) with arterial puncture within the last seven days; 5) with heparin or oral anticoagulants within the last 14 days, prolonged bleeding (>15s), platelet count <100×10^9^/L; 6) with hypoglycaemia with blood glucose <2.7 mmol/L, or diabetes with blood glucose >22.2 mmol/L; 7) with abdominal dialysis or hemodialysis; 8) with serious heart, liver, or kidney dysfunction; 9) with malignant tumor or ongoing anti-tumor therapy.

### Treatment of the patients

After confirmation of no bleeding or low density shadow by cranial CT, normal blood routines, no previous history of blood disorder or oral anticoagulant medication, or no serious history of hepatonephric disease, the patients in group A were given 0.6-0.7 mg/kg rt-PA and <30 minutes-DNT for the thrombolytic therapy without considering the results of blood clotting and hepatonephric function assay; the patients in group B were given 0.6-0.7 mg/kg rt-PA and 60-min DNT after received normal results of blood clotting and hepatonephric function assay; the patients in group C were given 0.9 mg/kg rt-PA and 60-min DNT after received normal results of blood clotting and hepatonephric function assay.

rt-PA (Boehringer Ingelheim Co., Ltd., Germany) was administrated with the dose as 0.6 mg/kg (the maximum amount 50 mg) or 0.9 mg/kg (the maximum amount 90 mg); 10% of the total dose was performed rapid intravenous bolus, lasting one minute; then the rest 90% was prepared into injections and continuously administered through intravenous infusion pump, lasting one hour; reviewed the patients’ cranial CT one day after the thrombolytic therapy, and administrated the patients with 100 mg/d aspirin after excluded the incidence of intracranial hemorrhage.

### Observation indexes

The NIHSS scores of the three groups before the thrombolytic therapy as well as at H1, H24, D7, D30, and D90 after the treatment were calculated to compare the clinical efficacy among the three groups. The post-treatment intracranial hemorrhage and mortality were compared to evaluate the safety of these three treatment regimens. The modified Rankin Scale (mRS) scores of the three groups at D90 were used for the prognostic evaluation, with mRS 0-1 point for good prognosis while mRS 2-5 points for poor prognosis.

### Statistical analysis

The clinical data were analyzed using SPSS18.0 software, and the intergroup difference was compared using the t test; the data were analyzed using the χ^2^ test; each factor was expressed as cases or percentage, with *P*<0.05 considered as statistically different.

## RESULTS

### General information

The 220 AIS patients were randomly divided into group A (90 cases), group B (90 cases), and group C (40 cases) with the premise of their own or their families’ signed informed consent, including 139 males and 81 females; aged 37-85 years, with the mean age as 62.2±11.8 years; the NIHSS scores when admitted were 4-22 points, with an average as 11.2±4.5. Previous disease history: 154 patients for hypertension, 46 patients for diabetes; 91 patient for stroke, 78 patients for organic heart disease, 27 patients for atrial fibrillation, 106 patients for hyperlipidemia, 93 patients for smoking; and 73 patients for the administration of anti-platelet drugs. The specific factors of the three groups were shown in Table-I.

**Table-I:** General information of the three groups.

Factor	A	B	C	χ^2^	P
Patient number	90 cases	90 cases	40 cases		
Age	62.7±13.1	61.2±10.7	63.5±12.2	0.337	0.845
Gender (Male/Female)	58 / 32	55 / 35	26 / 14	0.284	0.867
Weight (kg)	68.5±14.1	65.3±12.8	66.2±15.1	2.233	0.327
NIHSS score when admitted	11.4±4.3	10.9±3.8	11.5±5.5	0.273	0.872
Systolic blood pressure (mmHg)	153.2±16.8	149.7±20.5	151.6±22.4	1.516	0.469
Diastolic blood pressure (mmHg)	87.8±13.7	84.4±11.8	85.3±15.8	0.478	0.788
Period of receiving rt-PA (min)	195.8±62.3	236.7±70.6	240.3±68.1	8.647	0.013
Hypertension [n (%)]	64(71.1)	59(65.6)	31(77.5)	1.971	0.373
Diabetes [n (%)]	16(17.8)	21(23.3)	9(22.5)	0.915	0.633
Stroke [n (%)]	34(37.8)	44(48.9)	13(32.5)	3.874	0.144
Organic heart disease [n (%)]	38(42.2)	29(32.2)	11(27.5)	3.318	0.190
Atrial fibrillation [n (%)]	12(13.3)	8(8.9)	7(17.5)	2.066	0.356
Hyperlipidemia [n (%)]	42(46.7)	47(52.2)	17(42.5)	1.188	0.552
Smoking [n (%)]	35(38.9)	42(46.7)	16(40)	1.219	0.544
Administration of antiplatelet drug [n (%)]	21(23.3)	38(42.2)	14(35)	7.314	0.026

### NIHSS scores

**Fig.1:**
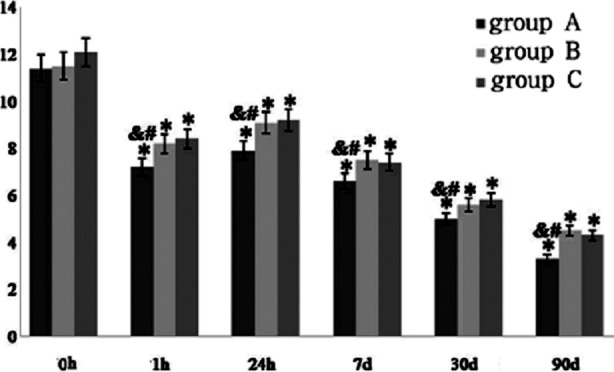
NIHSS scores of the three groups before and after the treatment; *compared with those before the treatment, P<0.05; ^&^compared with group B after the treatment, P<0.05; ^#^compared with group C after the treatment, P<0.05.

The comparisons of the NIHSS scores of the three groups before the treatment and at H1, H24, D7, D30, and D90 after the treatment revealed that the neurological functions of the three groups were all effectively improved (Fig.1). Compared with the NIHSS score before the treatment, those at H1, H24, D7, D30, and D90 after the treatment were all significantly reduced, and the differences were significant (*P*<0.05); and the NIHSS scores exhibited the significantly decreasing trend with the prolonged treatment time. Compared with group B and C, the NIHSS scores of group A at H1, H24, D7, D30, and D90 after the treatment were lower (*P*<0.05); while the differences between group B and C were not significant (*P*>0.05), indicating that the efficacies of the hypernormal shortened DNT thrombolytic treatment were significantly better than the conventional thrombolytic programs, and the thrombolytic effects of low-dose alteplase showed no difference with those required by the conventional thrombolytic programs.

### Safety and prognosis evaluation

Through analyzing the cases of gingival bleeding, gastrointestinal bleeding, cerebral bleeding, and death within 90 days after the treatment, the statistics of safety evaluation showed that the incidence of post-thrombolysis complications in the three groups were low, and the intergroup difference was not significant (*P*>0.05), indicating that these three treatment programs all had high clinical safety (Table-II).

**Table-II:** Safety of these three treatment programs after the treatment.

Group	Gingival bleeding	Gastrointestinal bleeding	Cerebral bleeding	Death within 90 days after the treatment
A	6.67%	0.00%	0.00%	2.22%
B	7.78%	1.11%	0.00%	3.33%
C	7.50%	0.00%	0.00%	0.00%
χ^2^	0.086	1.451	0	1.387
*P*	0.958	0.484	1	0.500

**Fig.2:**
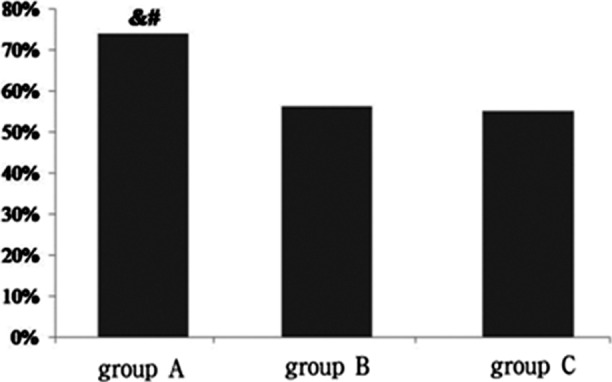
Rates of good prognosis of the three groups 90 days after the treatment; ^&^compared with group B after the treatment, P<0.05; ^#^compared with group C after the treatment, P<0.05

The mRS scores of the three groups 90 days after the treatment showed that group A had 65 cases evaluated as good prognosis, group B had 49 cases, and group C had 22 cases (Fig.2). The prognosis of group A was significantly better than group B and C, and the intergroup differences were significant (PAB=0.015, PAC=0.034); however, the difference between group B and C was not significant (PBC=0.889).

## DISCUSSION

Since the main risk of thrombolytic therapy for ischemic stroke is bleeding transformation, which is primarily related to the dosage and medication time; therefore, under the premise of ensuring the thrombolytic effects, the medication waiting time and the amount of alteplase should be reduced as much as possible, thus minimizing the risk of bleeding. This study mainly observed how to shorten the pre-treatment inspection time, alteplase dosage, and the best treatment time, etc. Through this study, we found that despite the huge divergence about the best medication time among different countries currently, for example, the application time of alteplase recommended by Europe and USA for AIS was inconsistent,^13^,^14^ there is no doubt that “time is the brain”, the shorter the interval between the onset and the initiation of thrombolytic therapy, the greater the benefits that the AIS patients might possibly obtain.^15^-^18^ Usually, the inspections of cranial CT, blood routine, blood clotting, blood glucose, and electrocardiogram (ECG) when patient admitted needed more than 60 minutes, especially the blood clotting and the hepatonephric function test might need at least 45 minutes. The patients in group A showed no bleeding or low density shadow in cranial CT, and the results of blood sugar, ECG, and blood routine test were normal. Under the premise that the patients’ family members provided the disease history (no previous history of blood disorder, oral anticoagulant medication, or serious hepatonephric disease), the patients in group A were directly administered the rt-PA thrombolytic therapy without the results of blood clotting and hepatonephric function test, which saved at least 30 minutes and greatly shorten the interval between the onset to the thrombolytic therapy; therefore, more patients could be applied the thrombolytic therapy.^19^ When the results of blood clotting were returned, the patients with normal test results could continue the thrombolytic therapy while those with obvious abnormal results treatment may be stopped. The results of blood clotting in Group-A showed no abnormal; therefore, it could be seen that the efficacies and prognosis in Group-A were better than those in group B and C (*P*<0.05), and the safety and mortality showed no significant difference than group B and C (*P*>0.05). Although it might have certain risk to begin the thrombolytic therapy without the results of blood clotting and hepatonephric function test, the premise was to preliminarily grasp the individual conditions of each patient in details, which could not only effectively reduce the risk but also start the treatment as soon as possible and increase the benefit rate. As for the point of improving the green channel mechanism towards the AIS treatment, the individualized hypernormal shortened DNT reduced the time interval from the onset of the symptoms to the application of the thrombolytic therapy. Therefore, it proposed a new way of thinking and a new attempt for the thrombolytic preparation work against stroke in future, and could provide ideas to develop expert consensus in future.

### Issue of the thrombolytic doses

The guidelines of US and European ischemic stroke treatment recommended 0.9 mg/kg as the standard therapeutic dose of rt-PA, and that of Japan was 0.6 mg/kg.^20^,^21^ Currently, China still lacks the relevant large-scale evidence-based medical researches, and in viewing the differences in race, physical conditions, and stroke risk factors between Chinese people and Westerners,^22^ whether 0.9 mg/kg is fully suitable still remains controversial among Chinese investigators. This study compared the efficacy, prognosis, and safety between group B and C, and the differences were not statistically significant (*P*>0.05), indicating that low-dose rt-PA thrombolytic therapy was safe and effective, and 0.6-0.7 mg/kg was likely to be the rt-PA dose suitable for Chinese people. Under the premise of ensuring the effectiveness, low-dose rt-PA thrombolytic therapy could not only reduce the risks of such high-dose treatment caused serious complications as gastrointestinal bleeding and cerebral bleeding but also reduce the economic burden of the patients.

In summary, the thrombolytic efficacy of the hypernormal shortened DNT plus low-dose alteplase were significantly better than the conventional therapies, which could greatly reduce the pre-medication waiting time, maximally expand the suitable populations, and increase the thrombolytic effects, and its efficacies were significantly better than the traditional method. The low-dose alteplase had the same thrombolytic effects as the conventional dose; furthermore, its safety and clinical utility were better, so the combination of these two could exhibit better effects. It was significantly better than the traditional methods which waited for the test results before the thrombolytic treatment, and the low-dose alteplase also exhibited the same thrombolytic effect as the conventional doses, so the safety was good.

## References

[1] Furie KL, Kasner SE, Adams RJ, Albers GW, Bush RL, Fagan SC (2011). Guidelines for the prevention of stroke in patients with stroke or transient ischemic attack: a guideline for healthcare professionals from the american heart association/american stroke association. Stroke.

[2] Saver JL, Albers GW, Dunn B, Johnston KC, Fisher M, STAIR VI Consortium (2009). Stroke Therapy Academic Industry Roundtable (STAIR) recommendations for extended window acute stroke therapy trials. Stroke.

[3] Brekenfeld C, Schroth G, Mattle HP, Do DD, Remonda L, Mordasini P (2009). Stent placement in acute cerebral artery occlusion: use of a self-expandable intracranial stent for acute stroke treatment. Stroke.

[4] Addo J, Ayerbe L, Mohan KM, Crichton S, Sheldenkar A, Chen R (2012). Socioeconomic status and stroke: an updated review. Stroke.

[5] Bejot Y, Catteau A, Caillier M, Rouaud O, Durier J, Marie C (2008). Trends in incidence, risk factors, and survival in symptomatic lacunar stroke in Dijon, France, from 1989 to 2006: a population-based study. Stroke.

[6] Charidimou A, Kakar P, Fox Z, Werring DJ (2013). Cerebral microbleeds and the risk of intracerebral haemorrhage after thrombolysis for acute ischaemic stroke: systematic review and meta-analysis. J Neurol Neurosurg Psychiatry.

[7] Wahlgren N, Ahmed N, Dávalos A, Hacke W, Millán M, Muir K (2008). Thrombolysis with alteplase 3-4.5h after acute ischaemic stroke (SITS-ISTR): an observational study. Lancet.

[8] Wardlaw JM, Murray V, Berge E, del Zoppo G, Sandercock P, Lindley RL (2012). Recombinant tissue plasminogen activator for acute ischaemic stroke: an updated systematic review and meta-analysis. Lancet.

[9] Wardlaw JM, Murray V, Berge E, del Zoppo GJ (2009). Thrombolysis for acute ischaemic stroke. Cochrane Database Syst Rev.

[10] Ding S, Wang T, Cui W, Haydon PG (2009). Photothrombosis ischemia stimulates a sustained astrocytic Ca^2+^ signaling in vivo. Glia.

[11] Hacke W, Kaste M, Bluhmki E, Brozman M, Dávalos A, Guidetti D (2008). Thrombolysis with alteplase 3 to 4.5 hours after acute ischemic stroke. N Engl J Med.

[12] Adeoye O, Knight WA, Khoury J, Schmit PA, Sucharew H, Broderick JP (2014). A matched comparison of eptifibatide plus rt-PA versus rt-PA alone in acute ischemic stroke. J Stroke Cerebrovasc Dis.

[13] European Stroke Organisation (ESO) Executive Committee (2008). ESO Writing Committee, Guidelines for management of ischaemic stroke and transient ischaemic attack 2008. Cerebrovasc Dis.

[14] Minematsu K, Toyoda K, Hirano T, Kimura K, Kondo R, Mori E (2013). Guidelines for the intravenous application of recombinant tissue-type plasminogen activator (alteplase), the second edition, October 2012: a guideline from the Japan Stroke Society. J Stroke Cerebrovasc.

[15] Hacke W, Donnan G, Fieschi C, Kaste M, von Kummer R, Broderick JP (2004). Association of outcome with early stroke treatment: pooled analysis of ATLANTIS, ECASS, and NINDS rt-PA stroke trials. Lancet.

[16] Saver JL (2006). Time is brain--quantified. Stroke.

[17] Emberson J, Lees KR, Lyden P, Blackwell L, Albers G, Bluhmki E (2014). Effect of treatment delay, age, and stroke severity on the effects of intravenous thrombolysis with alteplase for acute ischaemic stroke: a meta-analysis of individual patient data from randomised trials. Lancet.

[18] Lees KR, Bluhmki E, von Kummer R, Brott TG, Toni D, Grotta JC (2010). Time to treatment with intravenous alteplase and outcome in stroke: an updated pooled analysis of ECASS, ATLANTIS, NINDS, and EPITHET trials. Lancet.

[19] Meretoja A, Strbian D, Mustanoja S, Tatlisumak T, Lindsberg PJ, Kaste M (2012). Reducing in-hospital delay to 20 minutes in stroke thrombolysis. Neurology.

[20] Adams H, Adams R, Del Zoppo G, Goldstein LB (2005). Stroke Council of the American Heart Association;American Stroke Association. Guidelines for the early management of patients with ischemic stroke:2005 guidelines update a scientific statement from the Stroke Council of the American Heart Association/American Stroke Association. Stroke.

[21] Yamaguchi T, Mori E, Minematsu K, Nakagawara J, Hashi K, Saito I (2006). Alteplase at 0.6 mg/kg for acute ischemic stroke within 3 hours of onset. Japan Alteplase Clinical Trial (J-ACT). Stroke.

[22] Breuer L, Nowe T, Huttner HB, Blinzler C, Kollmar R, Schellinger PD (2010). Weight approximation in stroke before thrombolysis: the WAIST-Study: a prospective observational “dose-finding” study. Stroke.

